# Annotation of the goat genome using next generation sequencing of microRNA expressed by the lactating mammary gland: comparison of three approaches

**DOI:** 10.1186/s12864-015-1471-y

**Published:** 2015-04-11

**Authors:** Lenha Mobuchon, Sylvain Marthey, Mekki Boussaha, Sandrine Le Guillou, Christine Leroux, Fabienne Le Provost

**Affiliations:** INRA, UMR1313 Génétique Animale et Biologie Intégrative, F-78350 Jouy-en-Josas, France; INRA, UMR1213 Herbivores, F-63122 Saint Genès Champanelle, France; Clermont Université, VetAgro Sup, UMR Herbivores, BP 10448, F-63000 Clermont-Ferrand, France

**Keywords:** miRNA, Goat, Mammary gland, Lactation, Deep sequencing, Chromosomal location, Host genes

## Abstract

**Background:**

MicroRNAs (miRNA) are small endogenous non-coding RNA involved in the post-transcriptional regulation of specific mRNA targets. The first whole goat genome sequence became available in 2013, with few annotations. Our goal was to establish a list of the miRNA expressed in the mammary gland of lactating goats, thus enabling implementation of the goat miRNA repertoire and considerably enriching annotation of the goat genome.

**Results:**

Here, we performed high throughput RNA sequencing on 10 lactating goat mammary glands. The bioinformatic detection of miRNA was carried out using miRDeep2 software. Three different methods were used to predict, quantify and annotate the sequenced reads. The first was a *de novo* approach based on the prediction of miRNA from the goat genome only. The second approach used bovine miRNA as an external reference whereas the last one used recently available goat miRNA. The three methods enabled the prediction and annotation of hundreds of miRNA, more than 95% were commonly identified. Using bovine miRNA, 1,178 distinct miRNA were detected, together with the annotation of 88 miRNA for which corresponding precursors could not be retrieved in the goat genome, and which were not detected using the *de novo* approach or with the use of goat miRNA. Each chromosomal coordinate of the precursors determined here were generated and depicted on a reference localisation map. Forty six goat miRNA clusters were also reported. The study revealed 263 precursors located in goat protein-coding genes, amongst which the location of 43 precursors was conserved between human, mouse and bovine, revealing potential new gene regulations in the goat mammary gland. Using the publicly available cattle QTL database, and cow precursors conserved in the goat and expressed in lactating mammary gland, 114 precursors were located within known QTL regions for milk production and composition.

**Conclusions:**

The results reported here represent the first major identification study on miRNA expressed in the goat mammary gland at peak lactation. The elements generated by this study will now be used as references to decipher the regulation of miRNA expression in the goat mammary gland and to clarify their involvement in the lactation process.

**Electronic supplementary material:**

The online version of this article (doi:10.1186/s12864-015-1471-y) contains supplementary material, which is available to authorized users.

## Background

MicroRNAs (miRNA) are small non-coding RNA that regulate targeted mRNA expression at a post-transcriptional level [1,2]. It is estimated that miRNA genes may account for 2-5% of all mammalian genes and regulate the expression of up to 60% of protein-coding genes [3,4]. miRNA play a major role in a broad range of biological processes. They are encoded in the genome and transcribed in a polII-dependent manner [5,6] as long transcripts from which long hairpin precursors are generated (pre-miRNA, ~70 nt) and cleaved out by the microprocessor Drosha endonuclease and cofactors [7]. The pre-miRNA are then exported to the cytosol where they are cleaved by the Dicer protein, releasing the loop and a duplex consisting of the mature -5p and -3p miRNA [7,8]. The miRNA are subsequently incorporated in the miRNA-induced silencing complex (miRISC) so that they can act on their targets.

Within a genome, a particular locus may carry several miRNA genes in what is called a cluster, defined as having a distance of less than 10 kb between each miRNA [9]. The expression of clustered miRNA is highly correlated, as they are often co-expressed from a single promoter as a single polycistronic transcript with neighbouring miRNA [10,11]. An internal coordination of clustered miRNA to regulate downstream biological networks has been suggested [12].

Approximately half of vertebrate miRNA are located in intergenic regions, referred to as intergenic miRNA [13,14]. The other half are intragenic, localized in introns and exons (about 40% and 10%, respectively) of protein-coding transcription units [14]. Intragenic miRNA tend to be co-expressed with their host genes [13]. Host genes and their resident miRNA have been considered to have synergistic effects [15,16]. Indeed, genes highly correlated in expression with an intragenic miRNA gene have been found to be more likely predicted as miRNA targets [17].

In the goat, the first publicly-available whole genome sequence (CHIR_1.0) was released in 2013 [18]. It consists of 30 pseudo-chromosomes (2.52 Gb) and an artificial chromosome designated as U (138 Mb) [18]. The high level of colinearity between goat and cattle chromosomes enabled assembly of the goat genome using the cattle genome as a reference. The annotation of potential goat protein-coding genes was performed using the homology-based annotation of proteins from cattle and humans [18].

Deep whole transcriptome sequencing, also known as RNA sequencing (RNA-seq), coupled with the development of several computational approaches, has offered numerous opportunities to discover and study the occurrence of miRNA across the genome [19,20]. Moreover, the availability of whole genome sequences has enabled the rapid annotation of miRNA. In the goat, some studies have been undertaken to determine miRNA in different tissues using this technology. The miRNA thus detected were firstly annotated using the sequences available in miRBase. For example, cattle and sheep miRNA from miRBase were used to annotate several miRNA in the skin of cashmere goats, and in the testis and mammary gland during both dry periods and at peak lactation [21-23]. Both ruminant and sheep genomes were also used to map the sequencing reads, as described by Ji *et al.* [19], who identified several miRNA in the goat mammary gland at early lactation. Furthermore, available Expressed Sequences Tags (EST) for the goat, combined with miRNA from numerous species (sheep, cow, pig, dog and horse) enabled the characterisation of hundreds of miRNA in goat ovaries and muscle [24,25]. Once the goat genome had been released, it started to be used for miRNA identification. Dong *et al.* [18] also identified 487 miRNA in their assembly using predictions by the INFERNAL software [26] against the Rfam database. Among the 487 miRNA found, 157 were located in 44 genomic clusters containing between two and 46 miRNA. Comparing the miRNA sequences from human, cattle, dog, chimpanzee, mouse and rat, they determined six goat-specific miRNA [18]. The goat genome was also utilized for the mapping of sequencing reads from goat testis, urine and skeletal muscle, and annotation of the miRNA thus characterized was performed by sequence homology with sheep, cow or human miRNA [27,28]. Finally, in order to map sequencing reads to the goat genome, miRDeep2 software combined with sequences from miRBase were used to detect 205 known and nine putative miRNA in goat hair follicles [29]. Although the goat genome has recently enabled the identification of several known and putative miRNA in different goat tissues as described above, it is still necessary to implement the reference list for goat miRNA, particularly in the lactating mammary gland, as much as decipher the involvement of miRNA in lactation which has recently begun to be investigated *in vitro* or in other species [30-33]. In the goat, a potential role for *miR-103* and *miR-27a* in the regulation of milk fat synthesis in mammary epithelial cells has been reported [34,35]. Despite these studies, the role of mammary miRNA is still poorly documented and greater knowledge of mammary goat miRNA will enable elucidation of the functions of miRNA in this organ.

Our increasing knowledge of the goat genome means it is now relevant to determine associations of marker alleles with phenotypes of interest in this species that are indicative of quantitative trait loci (QTL). In livestock, the detection of QTL is a step towards identifying genes and causal polymorphisms for traits of importance to agriculture and selection [36]. Most studies focused on detecting QTL for milk production have been carried out in cattle [37-40], and only preliminary studies have been performed in the goat [41], perhaps because of the lack of genomic tools for this species. A clearer understanding of miRNA and their localisation in the goat genome may offer perspectives to decipher the complexity of traits in dairy goats.

The work presented was therefore intended to generate an overview of miRNA expressed in lactating goat mammary gland. The precise location of their genes in the goat genome was determined by comparing three approaches to predict and annotate the sequencing reads. The localisation of miRNA precursors was compared in human, mouse and cattle in order to predict the genomic localisation of goat miRNA which might suggest conserved regulation in this species. Their genomic repartition and conservation across the 3 species mentioned above as well as their position relative to known bovine milk QTL, were investigated. Our findings offer new data on goat miRNA and genome annotation that will enable further studies in this species.

## Methods

### Animals and tissue sampling

All animal manipulations were performed in strict accordance with the guidelines of the Code for Methods and Welfare Considerations in Behavioral Research with Animals (Directive 86/609 EC) and the recommendations of the CEMEAA (Ethics Committee for Animal Experimentation in Auvergne). Every effort was made to minimize animal suffering. Ten peak-lactating Alpine goats (48 ± 2 d post-partum), from the INRA Experimental Farm in Lusignan (France) were slaughtered, and directly after death, ~50 g of mammary gland were collected under sterile conditions from the secretory area containing lobulo-alveolar structures (acini). The samples were frozen immediately in liquid nitrogen and stored at −80°C until RNA extraction.

### RNA isolation

Total RNA was extracted from ~150 mg mammary tissue using TRIZol® Reagent (Life Technologies) and further purified with the SV Total RNA Isolation system (Promega) to eliminate any contaminating genomic DNA. RNA purity and concentration were estimated by spectrophotometry (Nanodrop™, ND-1000) and using a 2100 Bioanalyzer Instrument (Agilent). Samples with a RNA Integrity Number (RIN) higher than 8, corresponding to high RNA quality, were used for the study.

### Library preparation and sequencing

Library preparation and sequencing was performed by the IGBMC Microarray and Sequencing Platform (Strasbourg, France). The TruSeq^TM^ small RNA kit protocol (Illumina) was followed to generate small RNA libraries directly from ~7 μg of total RNA, suitable for subsequent high throughput sequencing. Briefly, during the first step, RNA adapters were ligated sequentially to each end of the RNA; firstly the 3′ RNA adapter (5′ TGGAATTCTCGGGTGCCAAGG 3′) which is specifically designed to target microRNAs and other small RNAs containing a 3′ hydroxyl group resulting from enzymatic cleavage by Dicer or other RNA processing enzymes, and then the 5′ RNA adapter (5′ GTTCAGAGTTCTACAGTCCGACGATC 3′). Small RNA ligated with 3′ and 5′ RNA adapters were then reverse transcribed and PCR amplified (30 sec at 98°C; [10 sec at 98°C, 30 sec at 60°C, 15 sec at 72°C] × 13 cycles; 10 min at 72°C) to obtain cDNA. The final step was acrylamide gel purification of the 140–150 nt amplified cDNA (corresponding to cDNA obtained from small RNA + 120 nt from the adapters). The libraries were checked for quality and then quantified using the 2100 Bioanalyzer Instrument (Agilent). Libraries were loaded in the flowcell at an 8 pM concentration and clusters were generated using Cbot and sequenced on HiSeq 2500 (Illumina) as single-end 50 base reads, according to the manufacturer’s instructions. The quantity and quality of reads for each library are shown in Additional file 1: Table S1. RNA sequencing data were deposited in the Gene Expression Omnibus (GEO): GSE61025.

### Sequencing data processing

After removing sequences which corresponded to the sequencing adapters, and filtering by size (17–28 nt), using Cutadapt [42] data analyses were processed using miRDeep2 software [43], as described by Le Guillou *et al.* [44] and performed on the bioinformatics platform Genotoul (http://bioinfo.genotoul.fr/). The cleaned sequences were clustered into unique reads and then mapped to the reference goat genome (CHIR_1.0, [18]) using mapper.pl module. Putative miRNA and precursors were identified using the miRDeep2 core module, miRDeep2.pl (including reads corresponding to typical products of miRNA biogenesis, stability of the putative pre-miRNA hairpin and homology to previously identified miRNA). The miRDeep2 core algorithm needs to use known miRNA from the species under analysis, held in a reference database such as miRBase [45]. As few goat miRNA are reported in miRBase v21, three approaches were used. Thus, the use of the 2 miRNA from the virus BK Polyomavirus (*Bkv) (*precursor*: bkv-mir-B1*, mature: *bkv-miR-B1-5p* and *bkv-miR-B1-3p*), the 793 bovine miRNA (*Bos taurus, bta*), or the 436 goat miRNA from miRBase v21 were compared. Three independent sets of potential precursors (from BK Polyomavirus, bovine and goat) were created from the miRDeep2 prediction with a miRDeep2 score ≥0. Further, precursors from the BK Polyomavirus, bovine or goat containing known miRNA not found in potential precursors were added to the sets. The same operation was then performed to create three independent datasets containing putative miRNA and all known miRNA from miRBase v21. The quantifier.pl miRDeep2 module was then used to map unique reads and new sets of miRNA on new sets of potential precursors. The quantification results produced by the quantifier.pl module were then filtered with a custom perl script parse_miRDeep2_outputs.pl (https://mulcyber.toulouse.inra.fr/projects/bioinfoutils/) to eliminate any redundancy between known and putative miRNA. miRNA with at least ten read counts summing the ten libraries were considered, thus putative miRNA can be submitted to miRBase [46].

### Quantitative RT-PCR

Nine miRNA were chosen for RT-qPCR validation; miR-29a-3p (TaqMan® ID 007600_mat, Applied Biosystems), miR-99a-5p (TaqMan® ID 006254_mat), miR-126-3p (TaqMan® ID 008451_mat), miR-140-3p (TaqMan® ID 471823_mat), miR-222-3p (TaqMan® ID 000525), miR-223-3p (TaqMan® ID 002295), miR-204-5p (TaqMan® ID 000508), miR-409-3p (TaqMan® ID 002332), miR-6119-5p (Custom TaqMan® small RNA Assay). Reverse transcription was achieved on 10 ng of total RNA using the TaqMan® MicroRNA Reverse Transcription (Applied Biosystems, Foster City, CA, USA) kit following the manufacturer’s instructions. In the thermal cycler (StepOne+, Applied Biosystems, Foster City, CA, USA), each 15 μL RT reaction followed 30 min at 16°C, 30 min at 42°C, 5 min at 85°C. Then, 1.3 μL of miRNA-specific cDNA from the reaction were amplified using the TaqMan® Small RNA Assays (Applied Biosystems, Foster City, CA, USA) following the manufacturer’s instructions. Amplification was performed at 95°C for 10 min, pursued by 40 cycles of 95°C for 15 s and 60°C for 1 min. All miRNA levels were normalized to the values of U6 snoRNA [47,48].

### Screening for intragenic miRNA in the human, mouse, cattle and goat genomes

The chromosomal positions of miRNA in the human, mouse and cattle genomes were downloaded from miRBase v21 (http://mirbase.org/). Datasets for protein-coding genes were downloaded from BioMart Ensembl release 78, using the latest version of the genome (GRch38 for human, GRCm38.p3 for mouse and UMD3.1 for cattle (http://www.ensembl.org/biomart)). The positions of mRNA and the Coding Protein Sequence on the goat genome were obtained from Dong et al. [18]. Comparisons of the chromosomal locations of miRNA and protein-coding genes were performed using the IntersectBed tool in BEDTools software [49] with options requiring the same strandedness and an overlap of 100% necessary to cross miRNA and gene coordinates.

### Screening for miRNA in QTL

All Bovine QTL were downloaded from CattleQTLdb (AnimalQTLdb release 22, http://www.animalgenome.org). QTL traits linked to milk lactose, fat and protein content or yield and milk somatic cell scores, with a significance equal to significant and/or a p-value <0.05 were extracted from the entire bovine QTL file. Screening for mammary miRNA in the QTL was performed using IntersectBed with the options used to compare the chromosomal location of miRNA and protein-coding genes.

## Results and discussion

### Comparison of strategies for miRNA sequence identification

Next generation sequencing (NGS) technologies coupled with bioinformatic analysis offer a powerful method to analyze miRNA gene expression which allows for both the measurement of known miRNA and the identification of novel miRNA [50]. Unlike other technologies, they enable not only the discovery of novel miRNA but also the capacity to detect weakly expressed miRNA.

**Figure 1:**
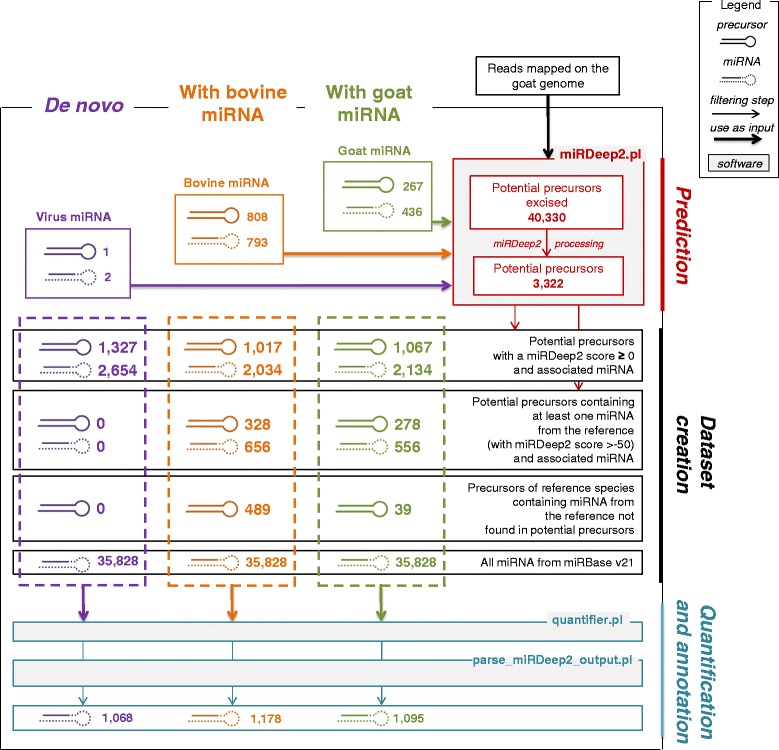
Summary of the three approaches used to predict, quantify and annotate sequencing reads. Common processes and results are indicated in red for the prediction step and in blue for the quantification and annotation steps. Specific processes and results are indicated in purple for the *de novo* approach, in orange and green for the approach using bovine and goat miRNA, respectively.

Here, we performed RNA-Seq on RNA isolated from the mammary gland of lactating goats, using Illumina/Solexa NGS technologies. Bioinformatic analyses of sequenced products were performed using miRDeep2 software which can identify known and putative miRNA with an accuracy of 98.6-99.9%, as reported by Friedländer *et al.* [43]. As in a previous study where the mammary gland miRNome of lactating cow was established [44], the miRDeep2 software was used to determine the miRNome of lactating goat mammary gland. As required by the miRDeep2 algorithm, both a list of known miRNA and a whole genome sequence are necessary for the species of interest in order to predict and quantify miRNA from the sequencing reads. During this study, and using the whole goat genome that has recently become available [18], three strategies (Figure 1) were compared for the prediction and annotation of miRNA. The first strategy was a *de novo* approach based on the use of BK Polyomavirus, a species that is phylogenetically distant from goat, with very few known miRNA (1 precursor and its 2 miRNA) were available in miRBase v21 and for which we checked that corresponding sequences could not be found in the goat genome. Thus this strategy was unable to retrieve viral miRNA in the goat genome and only managed to predict potential precursor from it. The second strategy used bovine precursors from miRBase v21, because bovine is a phylogenetically related species of the goat and has almost twice the number of goat miRNA. Finally, the third strategy was based on the use of goat precursors very recently available in miRBase v21.

#### Prediction of miRNA

The first step in the process was the prediction of precursors from the location of mapped reads on the goat genome using miRDeep2 (Figure 1). The miRDeep2 core algorithm scores each potential precursor excised from the goat genome principally for the combined compatibility of energy stability, positions and frequencies of reads with the Dicer processing signature [51]. The prediction results produced by miRDeep2 revealed the same number of potential precursors (3,322) with the three strategies (Figure 1). As a result, external information did not influence either precursor prediction or the associated scores.

**Figure 2:**
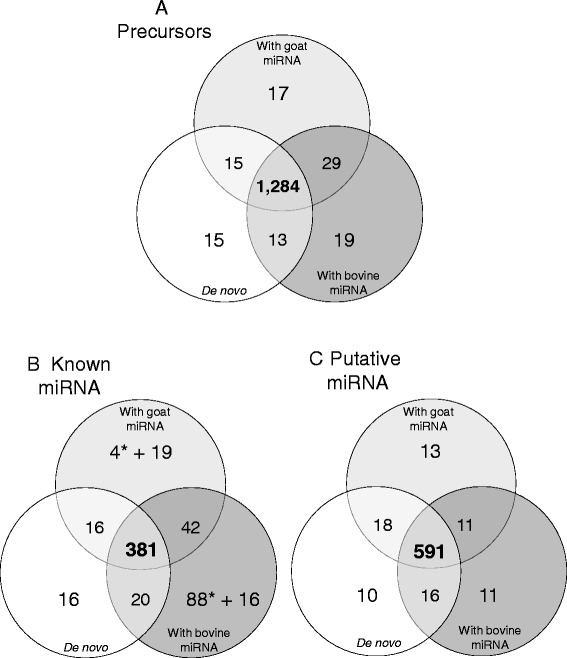
Comparison of the number of potential precursor and miRNA obtained by the three approaches. Potential precursors are represented in **A**, known and putative miRNA are represented in **B** and **C**, respectively. *: Adding precursors of reference species containing known miRNA not found initially in potential precursors enabled the annotation of 88 and 4 miRNA with the approach using bovine and goat miRNA, respectively.

Precursors with a miRDeep2 score ≥0 were retained as new potential precursors in this study. The three strategies produced different numbers of potential precursors with a miRDeep2 score ≥0. Thus 1,327 potential precursors were identified using the *de novo* approach, while 1,017 and 1,067 potential precursors were predicted using the bovine and goat miRNA-based strategy, respectively (Figure 1). These differences were due to 328 and 278 potential precursors containing mature miRNA known in bovine and goat respectively, and referred to not as potential precursors but known precursors. Overall, we obtained 1,345 potential precursors using the bovine and goat approaches, of which 1,284 (95%) were common with the *de novo* approach (Figure 2).

The differences between the three analyses were due to dual causality. The first is the random component of the prediction, which constitutes the RandFold significance. Indeed, some potential precursors had a p-value close to the threshold (p ≤ 0.05), and this value varied at each prediction, directly impacting the associated miRDeep2 score (+3.8 for a significant p-value) and then put some precursors above or below the miRDeep2 threshold we fixed. The second difference between analyses only affected potential precursors which contains known miRNA of the reference species. Actually, some miRNA were known only in bovine (for instance *miR-1260b*) or in goat (for instance *miR-374e*), enabling the identification of specific potential precursors (15 in bovine and 4 in goat).

Our prediction results thus showed that the use of precursors from an external reference (i.e. bovine in this study) or miRNA for the studied species is not necessary to predict most of precursors from the genome (95%). Therefore, for a species with none or only a few miRNA in miRBase, a *de novo* prediction can produce results almost as complete as if precursors from an external or studied species were used.

For each approach, two sets of data were thus created: i) one containing potential precursors with or not known miRNA of the species, and ii) one containing putative miRNA and all known miRNA from miRBase v21 (Figure 1).

#### miRNA identification and quantification

Use of the quantifier.pl module and the custom script parse_miRDeep2_outputs.pl, as described by Le Guillou *et al.* [44], allowed us to map sequencing reads as well as the set of data containing miRNA (putative and known) on the data set containing potential precursors. Only precursors carrying miRNA with at least ten counts summing all libraries were retained reducing the numbers of potential precursors to 913 in the *de novo* approach, 924 with bovine and goat miRNA (Table 1, ‘Filtered’). In addition, depending on the location of reads relative to the position of known miRNA from miRBase on the “potential precursors”, reads were assigned to miRNA, and two miRNA (5p and 3p) were retained for each precursor. This step enabled the quantification and annotation of 1,068, 1,178 and 1,095 distinct miRNA using the *de novo*, bovine or goat miRNA approaches, respectively; these included both known and putative miRNA which had not previously been described in any species from miRBase v21. The quantifier.pl module from miRDeep2 provided secondary structures of precursors containing known or putative miRNA and few examples are exposed in Additional file 2: Figure S1. Then, among the common miRNA obtained using the three methods, 381 were known miRNA and 591 were putative miRNA (Figure 2). As different precursors produce identical miRNA, the number of miRNA annotated here was not the double of the number of precursors. This similar annotation of mature miRNA was not surprising regarding these miRNA because of the strong similarity between sets of “potential precursors”. We also observed few putative miRNA that were only found using one or other of the approaches, corresponding to potential precursors with a random score ≥0 (Figure 2).

**Table 1:** **Comparison of precursors’ annotation results using the three approaches**

		**De novo**	**Bovine**	**Goat**
Potential precursors	Reads mapped (%)*	***75.1***	***74.9***	***74.9***
	**Total**	**1,327**	**1,017**	**1,067**
	Filtered** :	**913**	**924**	**924**
	**miRNA -5p and -3p**	**305**	**299**	**305**
	−5p + −3p annotated	172	172	172
	−5p annotated + −3p putative	20	20	20
	−5p putative + −3p annotated	14	13	14
	−5p + −3p putative	99	94	99
	**Only -5p**	**298**	**303**	**305**
	−5p annotated	53	59	59
	−5p putative	245	244	243
	**Only -3p**	**310**	**322**	**317**
	−3p annotated	60	67	66
	−3p putative	250	255	251
Known precursors	Reads mapped (%)*	***0***	***2.9***	***2***
	**Total**	**1**	**808**	**267**
	Filtered**:	**0**	**138**	**25**
	−5p + −3p known	0	4	8
	Only -5p known	0	55	8
	Only -3p known	0	49	8
	−5p known + −3p annotated	0	17	0
	−3p known + −5p annotated	0	13	1

Annotated miRNA correspond to miRNA annotated by homology (perfect match full length) with mature miRNA from miRBase v21. Putative miRNA correspond to miRNA not yet described in any species. In bold, numbers correspond to the total of each subcategory.

*Percentage of reads mapped on the set of potential precursors.

**Only potential precursors with cumulative counts >10 have been retained.

In addition, using the bovine or goat miRNA method, 489 bovine or 39 goat precursors (Figure 1) were added to the set of “potential precursors”, in which 138 and 25 precursors (Table 1, category of ‘known precursors’) enabled the supplementary quantification and annotation of 88 and 4 miRNA in bovine and goat approaches, respectively (Table 1, Figure 2). It appears that these miRNA corresponded to precursors not detected as potential precursors predicted by miRDeep2 at the prediction step. Therefore, the associated genome regions containing these miRNA may not be available in the actual version of the goat genome. Our results indicate that using an external reference combined with a good quality reference genome could produce a list of the miRNA expressed in a specific tissue. Indeed, the use of bovine precursors enabled the quantification of 88 more miRNA when compared with the *de novo* approach or using goat miRNA, giving an overview of the miRNA expressed in the lactating goat mammary gland. The use of bovine miRNA was also able to provide an assessment of the quality of the goat genome assembly, as this was only assembled very recently in its first version, CHIR_1.0, which might not be totally complete. Dong *et al.* [18] specified that around 89% of their raw paired-end sequences mapped to the assembled goat genome, suggesting that additional miRNA might still be found in the remaining 11%. Consequently, the 88 miRNA identified using bovine precursors may contribute to improving our knowledge of the genome.

Furthermore, our results pointed to the fact that about 23% of total reads did not map on the set of “potential precursors” (Table 1). They may correspond to miRNA that are not found in the goat genome or in bovine precursors. As an example, sequences with at least 500 summing reads in the 10 libraries may have represented 60% of unmapped reads corresponding to 445 unique sequences. To clarify the origin of these unmapped reads, a blast against all miRNA from miRBase v21 (one mismatch or gap allowed) was performed. Two sequences corresponding to miR-143 represented 20% of unmapped reads (Additional file 3: Table S2). Taken together, 88 of these unique sequences corresponded to 40 distinct miRNA, at least 37 of which are known in the bovine and needed to be taken into account during quantification. For example, a precursor containing mature miR-143 was predicted in the goat (chr7_10657), but the sequence of the potential precursor reported by miRDeep2 stopped at the end of the predicted mature miRNA. According to Friedländer *et al.* [43], the precursor sequence is the consensus precursor miRNA sequence inferred from deep sequencing reads. It represents the Drosha hairpin product, and does not include a substantial flanking genomic sequence, unlike most miRBase precursors. The absence of these flanking regions prevented mapping of these reads on potential precursors (reads mapping on “potential precursors” allowed 0 mismatches). The same scenario was hypothesized for the 36 miRNA known in bovine.

The combination of the tools used (quantifier.pl, parse_mirdeep2_outputs.pl, mature miRNA from miRBase) was sufficiently powerful to annotate potential precursors. Therefore, in order to produce the most complete annotation of the goat genome, the results of the approach using bovine miRNA were retained for the remainder of the analysis. The goat mammary gland miRNome established here thus comprised 1,178 mature miRNA, divided into 629 known and 549 putative miRNA (Additional file 4: Table S3, Additional file 5: Table S4). The expressions of nine miRNA were confirmed using RT-qPCR (Additional file 6: Figure S2).

### Annotation of the goat genome

**Figure 3:**
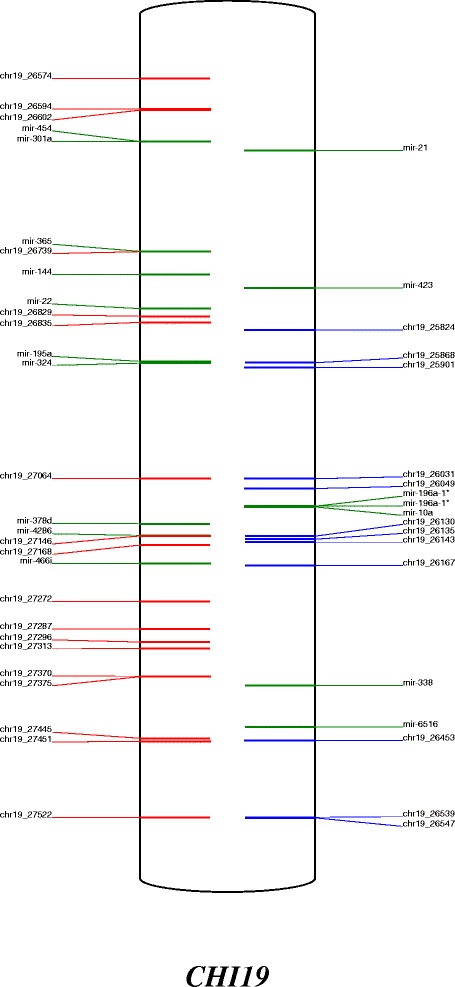
Map of the CHI 19 location of miRNA precursors. In left, putative (red) and known (green) miRNA localized on the + strand. In right, putative (blue) and known (green) miRNA localized on the - strand.

**Figure 4:**
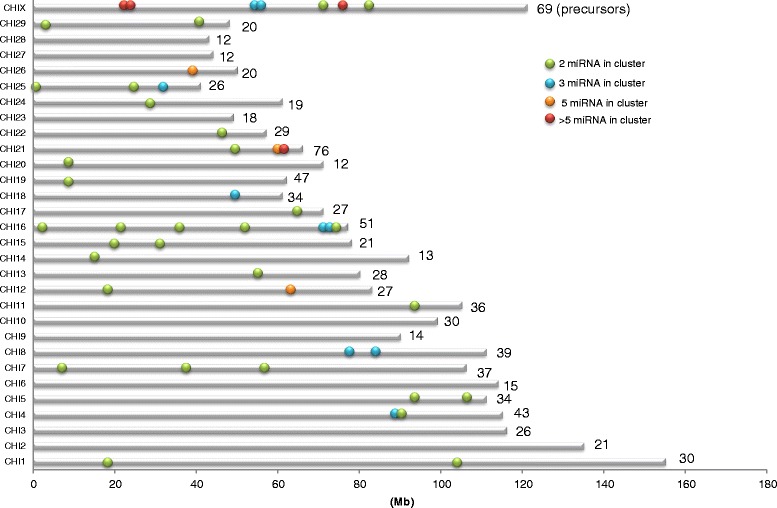
Number of precursors and clusters in each goat chromosome. Clusters were positioned on chromosomes. Interdistance between precursors of less than 10 kb was considered to be a cluster. The number of precursors per chromosome is indicated at the extremity of each chromosome.

An overview of precursors and miRNA expressed in the goat mammary gland at peak lactation was thus produced and each precursor was positioned on goat chromosomes (Figures 3, 4 and Additional file 7: Figure S3, Additional file 4: Table S3). The example of CHI 19 is shown in Figure 3. The 924 precursors were distributed throughout the chromosomes. Those with the most precursors were CHI 21, CHI X and CHI 19, having 76, 69 and 47 miRNA genes, respectively. Our results agreed with the chromosomal distribution of miRNA identified in previous reports [18,22], although we were able to provide a longer list of precursors representing around 2-fold of those previously described (487 and 464 locations identified by Dong *et al.* [18] and Wu *et al.* [22], respectively, versus 924 in the current study). Goat and bovine chromosomes have a high colinearity with those containing the most precursors being BTA 21, BTA X, and BTA 19 [52]. In addition, chromosome length does not correlate to the number of precursors, as previously observed by Ghorai and Ghosh [53]. Thus, for example, one of the longest goat chromosomes is CHI 2 (135 Mb) which only carries 21 precursors, while CHI 21 (63 Mb) carries 76 precursors (Figure 4). Density defined as the number of precursors/chromosome size is lower for CHI 6 and CHI 14 with a density of 0.13 and 0.14 respectively, while CHI 21 has a higher density of 1.13. However, chromosome X in several mammalian species has been reported to carry a higher density of miRNA precursors than those associated with autosomes in the testis, while precursors linked to the X chromosome have higher substitution rates than miRNA linked to autosomal chromosomes [54]. However, in the current instance, CHI X had a density of 0.57, representing the chromosome with the fifth highest density of precursors.

Because of the high colinearity between the goat and bovine genomes [18] most of the precursors identified during our study were located at identical positions in both species. However, the 21 members’ *mir-2284*, a ruminant-specific miRNA family [55], were not located on the same chromosome in the bovine and goat genomes, except for four precursors (*mir-2284ab*, *mir-2285 k*, *mir-2285 l* and *mir-2285o*). Furthermore, we used the publicly available genome to map our sequencing reads, containing chromosomes, scaffold and contigs. We were thus able to identify some precursors that were identical between chromosomes and contigs; for example, *mir-2284y* was retrieved on both. The high sequence homology between miRNA produced by many *mir-2284* family precursors may explain why they could be retrieved at several chromosomal locations and elucidate the difference in chromosomal positions between goat and bovine. This family was not yet fully described in goat although ruminant-specific miRNA may contribute to understand specific features of those species, especially in terms of lactation.

The goat genome contains large numbers of ruminant-specific repeat regions that account for 42.2% of the genome [18]. This can be perceived through miRNA annotation, because we identified several identical precursors with different locations. For example, on CHI 16, 2 precursors were predicted to produce *miR-29c*. Otherwise, a particular situation was observed because 29 overlapping precursors were detected on the goat genome. Indeed, at the same chromosomal location, one precursor was predicted by miRDeep2 with associated miRNA -5p and -3p, and another was predicted from the -3p of the first precursor. It was possible that both might produce identical miRNA -3p and -5p. No other such example exists in miRBase v21. Because they produced identical mature miRNA, the same name was assigned to these overlapping precursors (Additional file 4: Tables S3, Additional file 8: Table S5, Additional file 9: Table S6, and Additional file 10: Table S7).

In animals, miRNA genes are often found to be clustered on the genome [56,57]. For this reason, precursors located in close proximity to each other were searched for, with an inter-miRNA distance smaller than 10 kb [9]. Our results revealed the presence of 46 clusters containing 165 precursors which represented 18% of all the precursors identified during our study, which was less than in the cow where clustered miRNA genes represent 26% of all known bovine miRNA (Figure 4, Additional file 8: Table S5) [52]. The number of cluster varies according to the species, since 36%, 46% and 47% of miRNA are found clustered in zebrafish, mouse and human, respectively [9,58]. Most of the precursor clusters reported here were discistronic, in the sense that they comprised two precursors, as is usually observed [12]. But larger clusters exist on the goat genome; for example, CHI 21 carries a cluster of 41 precursors, which appears to be conserved in mammals [18], close to a cluster on BTA 21 containing 47 precursors. Moreover, 7 clusters were only made up of putative precursors, the largest of which contained 5 putative precursors on CHI 26. Most miRNA genes within 50 kb of each other displayed highly correlated expression patterns, as they could be processed from a polycistronic primary transcript [11]. However, due to their complex maturation and degradation, Guo and Lu [59] showed that they might also display differences in their levels of expression. Furthermore, miRNA genes in a cluster may have a functional relationship *via* co-regulating or coordinated regulatory processes [60]. Clusters often contain members of different miRNA families, particularly in animal genomes [1]. In the present case, several members of the same family (such as the *let-7* family) were identified as being clustered, as well as the largest miRNA gene cluster localized on CHI 21 which contained 16 members of the mir-154 family. Unrelated miRNA in the same cluster are often assumed to have similar targeting properties [56]. Further studies are necessary to clearly understand how the expression of goat miRNA clusters is regulated.

### Intragenic goat miRNA and the conservation of their location among human, mouse and cow

Unlike the whole genome sequences of other livestock species, the goat genome has not yet been very well characterized. To date, almost 22,175 protein-coding genes have been annotated, based on the bovine and human genome annotation data available [18]. On the other hand, it has been estimated that up to half of all vertebrate miRNA are processed from introns of protein-coding genes, or from genes encoding for other non-coding RNA [13].

By investigating intragenic precursors in the goat genome, 322 precursors were found to be located in goat mRNA and CDS, representing about 35% of all the precursors identified during our study. However, miRNA that share the same strand orientation of their host genes have similar transcription mechanisms [1,61]. Thus, 263 precursors were detected in 217 goat mRNA and CDS regions (Additional file 9: Table S6) with the same orientation, representing about 28% of all the precursors identified during the study. Among the 263 goat intragenic precursors, 68 were known and 195 were putative precursors. The low percentage of intragenic precursors (28%), compared with the 50% quoted in the literature, might have been due to a lack of annotation for protein-coding genes on the goat genome. Genome annotation could therefore be improved by determining the localisation of miRNA.

Twenty one host genes contained 2 or more precursors, such as *MCM7* on CHI 25 and *GABRE* on CHI X, which contained a cluster of 3 precursors. As for host gene functions, some of these are major actors in development of the mammary gland; for example, members of the ErbB signaling pathway (*ERBB2)*, or members of the Wnt signaling pathway such as *RSPO2* [62,63]. Others host genes play a critical role in mammary metabolism, such as *VLDLR* (Very low density lipoprotein receptor), *SREBF2* (Sterol responsive element binding factor 2), or *AGPAT6* (1-Acylglycerol-3-phosphate O-acyltransferase 6) (Additional file 9: Table S6).

Interestingly, among the 217 host genes, the expression of 76 coding genes was confirmed in the mammary gland of the same goat using previous data from microarrays ([64]; GEO Series accession number GSE6380). According to these observations, only 35% of host genes were expressed in the mammary gland at peak lactation. This low percentage is likely due to a lack of completeness of the microarray. The difference observed might also be due to the difference in detection level of the techniques employed.

Taken together, a clearer understanding of intragenic goat miRNA may highlight potential new regulations of miRNA and gene expression, some of which may play critical roles in the lactation function.

**Figure 5:**
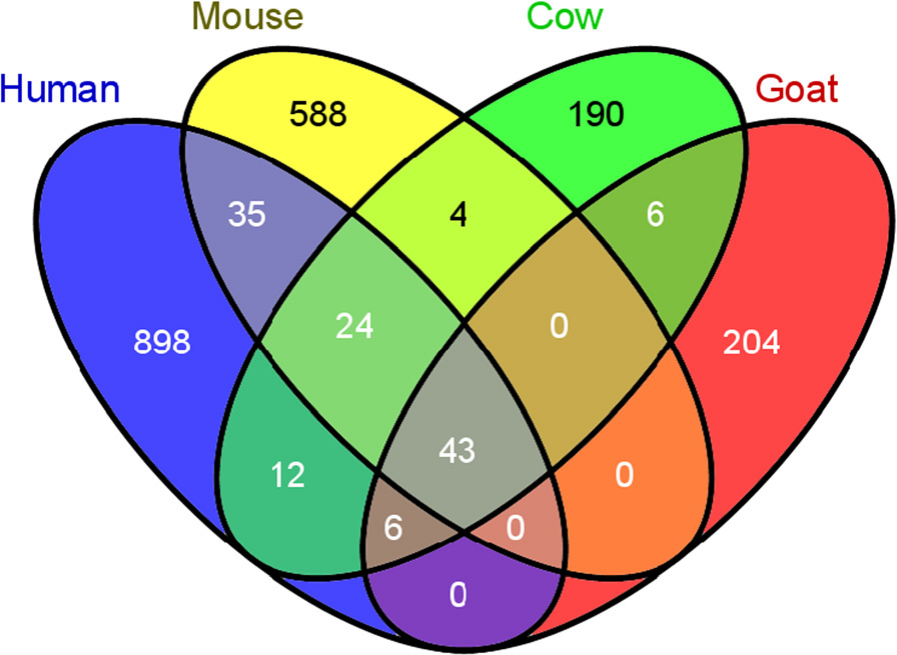
Conserved intragenic precursors in human, mouse, cow and goat. Human, mouse and cattle precursors were obtained from miRBase v21, and the locations of protein-coding genes were downloaded from BioMartEnsembl (http://www.ensembl.org/biomart). The locations of goat mRNA were obtained from Dong et al. [18].

Conservation of the location of miRNA/host genes throughout a species genome may offer a useful tool for its annotation. In human, mouse and cattle, 1,018, 694, and 285 precursors, respectively, in protein-coding genes have been detected as having the same orientation (Table 2). During the present study, a comparison of intragenic precursors in human, mouse and cattle with intragenic goat precursors revealed conservation of the location of 43 precursors (Figure 5, Additional file 10: Table S7). Due to the lack of annotation of goat protein-coding genes, we were not able to identify the precise location of conserved, intronic or exonic resident miRNA and compare it with conserved locations in the human, mouse or cow genomes. Godnic *et al.* [65] screened for intragenic precursors in the human, mouse and chicken genomes and found 27 precursors with conserved co-locations between the 3 species. Among these, 16 precursors were found amongst the 43 conserved between human, mouse, cow and goat in our analysis (*let-7 g, mir-101-2, mir-103, mir-107, mir-128-1, mir-1306, mir-140, mir-15b, mir-16b, mir-211, mir-218-1, mir-26a-1, mir-32, mir-33a, mir-455, let-7-2*), so the location of these precursors appears to be highly conserved in all vertebrates. Six intragenic precursors were found on chromosome X in human, mouse, cow and goat. Chromosome X was the only chromosome on which the locations of host genes were conserved between species compared using autosomes. The location of host genes on chromosome X appears to be markedly conserved between species (Additional file 10: Table S7). As some goat precursors were localized in genes coding for proteins for which the Ensembl name was not available, referred to as “deprecated identifiers” (Additional file 9: Table S6), the comparison with intragenic precursors in human, mouse and cow enabled the characterization of seven host genes per type of conservation (Additional file 9: Table S6). Furthermore, the locations of 24 precursors were conserved between human, mouse and cow (Figure 5). Among these 24 intragenic precursors, ten were detected in the goat during the present study. These ten miRNA could also be localized in protein-coding genes in the goat, but the lack of annotation of the goat genome probably prevented their identification. In addition, among these ten miRNA, *mir-128-2*, *mir-218-2* and *mir-301a* were found in the *ARPP21* (cAMP-regulated phosphoprotein), *SLIT3* (Slit homolog 3) and *SKA2* (Spindle and kinetochore associated complex subunit 2) genes in human, mouse, cow and chicken [65]. By investigating annotated goat mRNA, transcript coding for SKA2 was found to be annotated in the goat genome, suggesting that *mir-301a* is not intragenic in goat. However, the transcript coding for both ARPP21 and SLIT3 could not be retrieved. Furthermore, the genomes of human, mouse, cow and chicken have been well characterized by comparison with very recently assembled goat genome. Consequently, it could be hypothesized that *ARPP21* and *SLIT3* have not yet been described in the goat genome, and the presence of conserved intragenic precursors may indicate the location of these genes in this genome. Among the seven other conserved intragenic precursors in human, mouse and cow, *mir-1249* in the host gene *KIAA0930* and *mir-499* in the host gene *MYH7B* (Myosin heavy chain 7B cardiac muscle beta) were not retrieved either in the goat protein-coding genes available, suggesting once again that these genes have yet to be described in the goat genome. Using the conservation of precursor locations in well-known genomes may constitute a useful tool to predict the location of protein-coding genes that have not yet been described in newly assembled genomes such as the goat.

**Table 2:** **Intragenic miRNA precursors in human, mouse, cow and goat**

	**Human**	**Mouse**	**Cow**	**Goat**
Known miRNA genes	1,881	1,193	808	924
Within protein-coding genes	1,018	694	285	263
*% of known miRNA*	*54%*	*58%*	*35%*	*28%*

Human, mouse and cattle precursors were downloaded from miRBase v21, and protein-coding gene locations were downloaded from BioMartEnsembl (http://www.ensembl.org/biomart). The locations of goat mRNA were obtained from Dong et al. [18].

Evidence suggests that intragenic miRNA and host genes might be processed from the same RNA [66], and are often co-expressed with their host genes [13,67]. Previous studies indeed reported that conserved resident precursors such as *mir-26a/b* might cooperate with their host genes, the carboxy-terminal domain RNA polymerase II polypeptide A small phosphatase (*CTDSP*) family, in the regulatory network of G1/S phase transition [68]. Another example is *mir-33a*, which may act in concert with its *SREBF2* host genes to govern intracellular function and cholesterol homeostasis in vertebrates, thus representing an example of miRNA-host gene cooperation in regulating a metabolic pathway [69]. Evaluating the expression of host genes relative to that of their resident precursors in the lactating mammary gland could provide new insights into the regulation of mammary function and/or development.

### Screening for miRNA in bovine quantitative trait loci

Results of the QTL mapping studies that have been performed in livestock species are available in the animal QTL database (http://www.animalgenome.org/). Because no QTL mapping studies have been reported for goat in this QTL database, bovine QTL data were used in our study to investigate the localization of precursors expressed in mammary gland QTL. In cattle, some 2,307 QTL out of a total of 8,305 have been reported to be associated with milk traits (Additional file 11: Table S8).

Comparative mapping enables exploitation of the whole genome sequence and its annotation available for a model species, and inference of this information to other species whose genome annotation has not been well characterized. In this regard, a comparative analysis of the known goat precursors identified in the present study and the precursors expressed in bovine lactating mammary gland [44] was performed, and revealed 255 highly precursors conserved between these species.

Out of these, 114 were located within QTL regions associated with milk traits and distributed within all bovine chromosomes except BTA 1, 9, 11, 12, 17, 28 and X (Table 3). Cattle QTL have very large genome coverage and therefore could explain the anchorage of a high number of miRNA in these QTL regions. The presence of miRNA clusters in these QTL regions was also noted. Indeed, the larger cluster containing 42 precursors (*mir-379/656* cluster) in the bovine genome was found within a QTL region for milk fat percentage and content on BTA 21. The *mir-34b/34c* cluster, containing *mir-34b*, *mir-34c*, *mir-670*, *mir-129-2* and *mir-130a*, was also identified within QTL regions for milk fat yield and for milk protein yield on BTA 15.

**Table 3:** **Goat miRNA in milk bovine QTL**

	***Associated with production***	***Associated with components***				***Associated with health***
	**Milk yield**	**Milk fat**	**Milk protein**	**Milk cells**		
		***Milk fat percentage and content***	***Milk fat yield***	***Milk protein percentage and content***	***Milk protein yield***	***Somatic cell score***
**BTA2**						mir-26b
**BTA 3**	mir-186	mir-190b, mir-186	mir-190b, mir-186, mir-101-1	mir-190b,, mir-186, mir-101-1	mir-190b, mir-186	mir-190b,
**BTA 4**	mir-129-1	mir-490, mir-671, mir-196b, mir-148a, mir-335			mir-671, mir-148a, mir-196b	mir-671
**BTA 5**	mir-33a, let-7a-3, let-7b, mir-331, mir-26a-2, mir-1251, mir-135a-2	mir-33a, mir-331, mir-26a-2, mir-677, mir-135a-2, mir-1251	mir-331, mir-26a-2, mir-677, mir-135a-2, mir-1251	mir-33a, mir-331, mir-26a-2, mir-677, mir-135a-2, mir-1251	mir-331, mir-26a-2, mir-677, mir-135a-2, mir-1251	mir-331, mir-26a-2, mir-135a-2, mir-1251
**BTA 6**	mir-2284ab, mir-218-1	mir-2284ab, mir-218-1	mir-2284ab, mir-218-1	mir-2284ab, mir-218-1	mir-218-1	mir-2284ab, mir-218-1
**BTA 7**	mir-181c, mir-1271, mir-143, mir-145, mir-378a, mir-146a	mir-340, mir-1271, mir-145, mir-378a, mir-143, mir-146a	mir-340	mir-181c,	mir-181c, mir-143, mir-145, mir-378a, mir-146a	mir-181c, mir-143, mir-145, mir-378a, mir-146a
**BTA 8**					mir-455, mir-23b,, mir-24-1, let-7d	mir-31
**BTA 10**	mir-628		mir-628, mir-7859	mir-7859	mir-628, mir-7859	mir-628, mir-7859
**BTA 12**						
**BTA 13**	mir-499		mir-1388, mir-6123, mir-296, mir-499		mir-6123, mir-296, mir-499	
**BTA 14**	mir-151, mir-30d, mir-30b	mir-151, mir-30d, mir-30b,	mir-151, mir-30d, mir-30b	mir-151, mir-30d, mir-30b	mir-151, mir-30d, mir-30b	mir-151, mir-30d, mir-30b
**BTA 15**			mir-34b, mir-34c, mir-670, mir-129-2, mir-130a		mir-34b, mir-34c, mir-670, mir-129-2, mir-130a	
**BTA 16**	mir-34a				mir-34a, mir-29b-2, mir-29c	
**BTA 18**	let-7e, mir-125a, mir-138-2, mir-140, mir-99b		mir-769, mir-99b, let-7e, mir-125a	mir-99b, let-7e, mir-125a	mir-769, mir-99b, let-7e, mir-125a	mir-138-2, mir-140, mir-769, mir-99b, let-7e, mir-125a
**BTA 19**		mir-423, mir-196a-1, mir-10a, mir-338, mir-6516	mir-423, mir-196a-1, mir-10a, mir-338	, mir-423, mir-196a-1, mir-10a, mir-338		mir-423, mir-196a-1, mir-10a, mir-338
**BTA 20**	mir-449a, mir-582,	mir-582, mir-449a	mir-582	mir-582, mir-449a	mir-582, mir-449a	mir-582, mir-449a
**BTA 21**	mir-184, mir-7-1, mir-345, mir-127, mir-432, mir-136, mir-370, mir-379, mir-411a, mir-380, mir-411b, mir-758, mir-494, mir-543, mir-495, mir-376e, mir-376c, mir-376d, mir-376b, mir-376a, mir-1185, mir-381, mir-487b, mir-541, mir-655, mir-487a, mir-382, mir-134, mir-154a, mir-154b, mir-154c, mir-377, mir-541, mir-3957, mir-412, mir-369, mir-410, mir-656, mir-342, mir-382, mir-411c, mir-495, mir-665, mir-493, mir-485	mir-1185, mir-345, mir-127, mir-432, mir-136, mir-370, mir-379, mir-411a, mir-380, mir-411b, mir-758, mir-494, mir-543, mir-495, mir-376e, mir-376c, mir-376d, mir-376b, mir-376a, mir-1185, mir-381, mir-487b, mir-541, mir-655, mir-487a, mir-382, mir-134, mir-154a, mir-154b, mir-154c, mir-377, mir-541, mir-3957, mir-412, mir-369, mir-410, mir-656, mir-342, mir-382, mir-411c, mir-495, mir-665, mir-493, mir-485				mir-7-1
**BTA 22**			mir-135a-1, mir-191, mir-425	mir-26a-1, mir-138-1, mir-135a-1, let-7 g, mir-191, mir-425, mir-128-2		
**BTA 23**	mir-133b, mir-2285ad		mir-133b, mir-2285ad	mir-133b	mir-133b, mir-2285ad	mir-2285ad
**BTA 24**						mir-1-2, mir-133a-2
**BTA 25**	mir-106b, mir-25, mir-93				mir-106b, mir-25, mir-93	mir-193b
**BTA 26**	mir-146b, mir-202	mir-146b, mir-202	mir-146b, mir-202	mir-146b, mir-202	mir-146b, mir-202	
**BTA 27**	mir-383	mir-383			mir-383	
**BTA 29**	mir-194-2, mir-192				mir-708, mir-194-2, mir-192	

The names and locations of goat miRNA precursors identified during this study were compared with bovine miRNA expressed during lactation [44] and localized in QTL associated with milk (http://www.animalgenome.org).

Interestingly, several precursors were only found in QTL associated with one type of trait; for example, *mir-26b* on BTA 2 located in a QTL associated with the somatic cell score. On BTA 15, the 5 intraQTL precursors were found relative to milk fat and protein yield. Furthermore, on BTA 7, mir-340 was only found in QTL associated with milk fat. Finally, an intragenic miRNA, *mir-33a*, was also localized in a QTL linked to milk fat content. As mentioned above, the host gene of *mir-33a*, *SREBF2*, is known to regulate the expression of several lipogenic enzymes in numerous tissues involving the mammary gland, and plays a key role in controlling cholesterol homeostasis [70]. Intragenic *mir-33a* and the host gene *SREBF2* may act in a coordinated manner to govern lipid metabolism [69], their presence in QTL associated with milk fat content possibly revealing a role for this cooperation in the regulation of milk fatty acid traits. However, further investigations are needed to attribute different QTL to miRNA and their cooperation, because although the presence of precursors in QTL has been shown, no studies have yet attempted to unravel the role of miRNA in milk QTL traits.

Although QTL regions are not well conserved between breeds within the same species nor between different species, it is well documented that syntenic regions are highly conserved between species [71]. It is impossible at this stage to infer any association between these highly conserved goat miRNA genes and milk traits. However, syntenic regions may contain highly conserved orthologous genes and this information could constitute a starting point to study association of genes or clusters of genes with particular traits. Further validation studies should therefore be undertaken in order to check whether miRNA genes that are highly conserved in cattle could also be associated with milk traits.

## Conclusions

The present study provides a full catalogue of miRNA expressed in the goat mammary gland at peak lactation, together with each chromosomal location. To the best of our knowledge, this work represents a significant enrichment of the repertoire of goat miRNA and their location on the genome.

During this study, the bioinformatic detection of goat miRNA was carried out using three alternative strategies. The first one was a *de novo* predictive approach using the whole goat genome sequence for mapping and annotating sequenced reads. The second and third approaches involved a bovine and goat miRNA gene repertoire, respectively, as reference. Comparing the three approaches demonstrated that the second produced more exhaustive results, but *de novo* prediction revealed identical results that could be used as in a predictive and quantification strategy for species for which no or only a few miRNA have been reported in miRBase and whose genome is not fully known.

We report the identification of 924 miRNA in the goat mammary gland, 263 of which were found to be intragenic. Of these, the intragenic locations of 43 goat precursors were found to be conserved among human, mouse and cow, suggesting a conserved regulation of their expression between species regarding these intragenic miRNA. Conservation of the location of miRNA allowed us to hypothesize as to the location of genes that have not yet been annotated in the goat genome.

Preliminary studies to compare the goat and cattle genomes showed that 114 conserved precursors expressed in the lactating mammary gland of both species were localized within QTL regions associated with milk production traits. Further analyses are now required to clarify the potential effects of mammary miRNA on milk production traits, particularly in the goat.

All the goat miRNA identified during this work will be added to miRBase and can therefore serve as a reference for future studies.

### Availability of supporting data

The data sets supporting the results of this article are available in Gene Expression Omnibus (GEO) (http://www.ncbi.nlm.nih.gov/geo/) with accession number: GSE61025.

## Additional files

Additional file 1: Table S1. Read counts and percentage of read positions with an average base quality over 30. Additional file 2: Figure S1. Structure of some randomly chosen known and predicted precursors. In red associated miRNA. Additional file 3: Table S2. Sequences of unmapped reads on potential precursors. Additional file 4: Table S3. Chromosomal location of precursors on the goat genome. Additional file 5: Table S4. Goat miRNA expressed in the lactating mammary gland. Additional file 6: Figure S2. Quantitative RT-PCR validation of NGS data. Additional file 7: Figure S3. Map of miRNA precursors on the goat genome CHIR_1. At the left of the chromosome, putative (red) and known (green) precursors localized on the + strand,. At the right of the chromosome, putative (blue) and known (green) precursors localized on the – strand. Additional file 8: Table S5. Cluster of precursors in the goat genome. Additional file 9: Table S6. Intragenic goat precursors. Additional file 10: Table S7. Intragenic precursors conserved in human, mouse, cattle and goat. Additional file 11: Table S8. Bovine QTL associated with milk.
