# Supplementary figures and images for: Annotation of the goat genome using next generation sequencing of microRNA expressed by the lactating mammary gland: comparison of three approaches
Source: BMC Genomics. 2015 Apr 11;16(1):285. doi: 10.1186/s12864-015-1471-y (PMC4430871; doi:10.1186/s12864-015-1471-y)

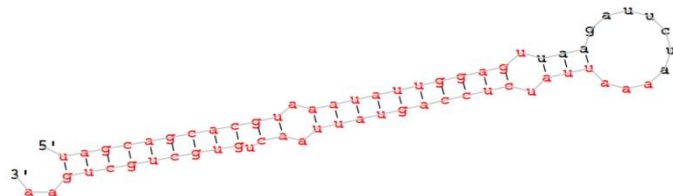

mir-16a

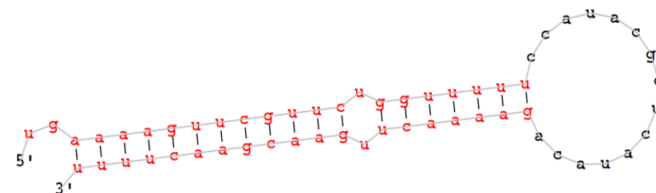

chr12\_12248

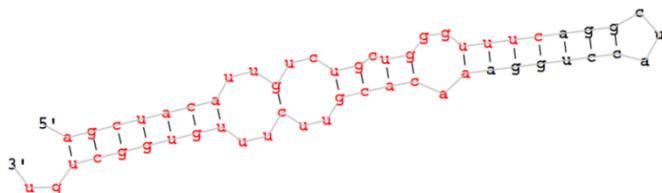

mir-221

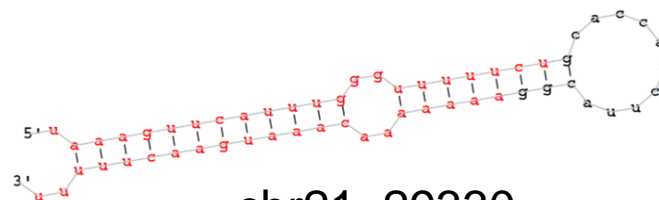

chr21\_29330

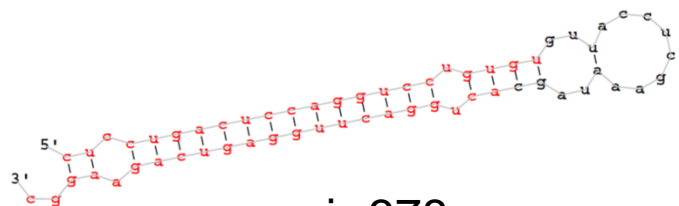

mir-378

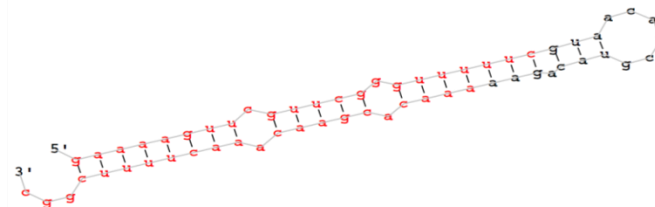

chr10\_15053

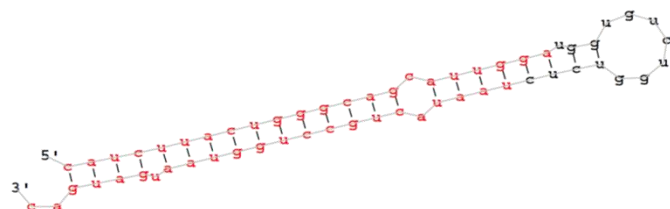

mir-200b

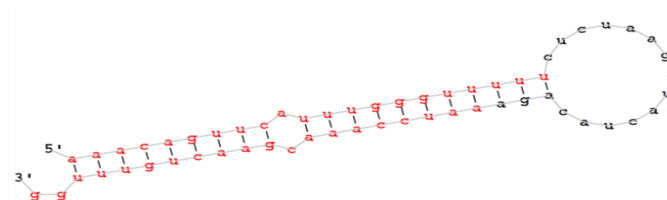

chr27\_34365

Known

Putative

Supplement: Additional file 2: Figure S1. — Structure of some randomly chosen known and predicted precursors. In red associated miRNA. [file 12864_2015_1471_MOESM2_ESM.pdf]

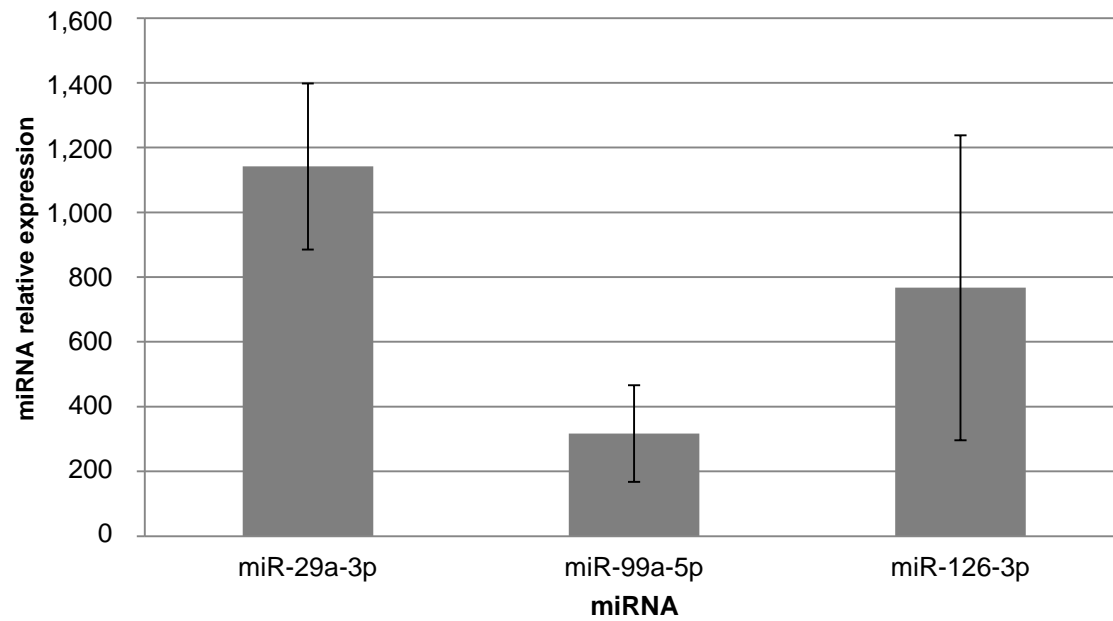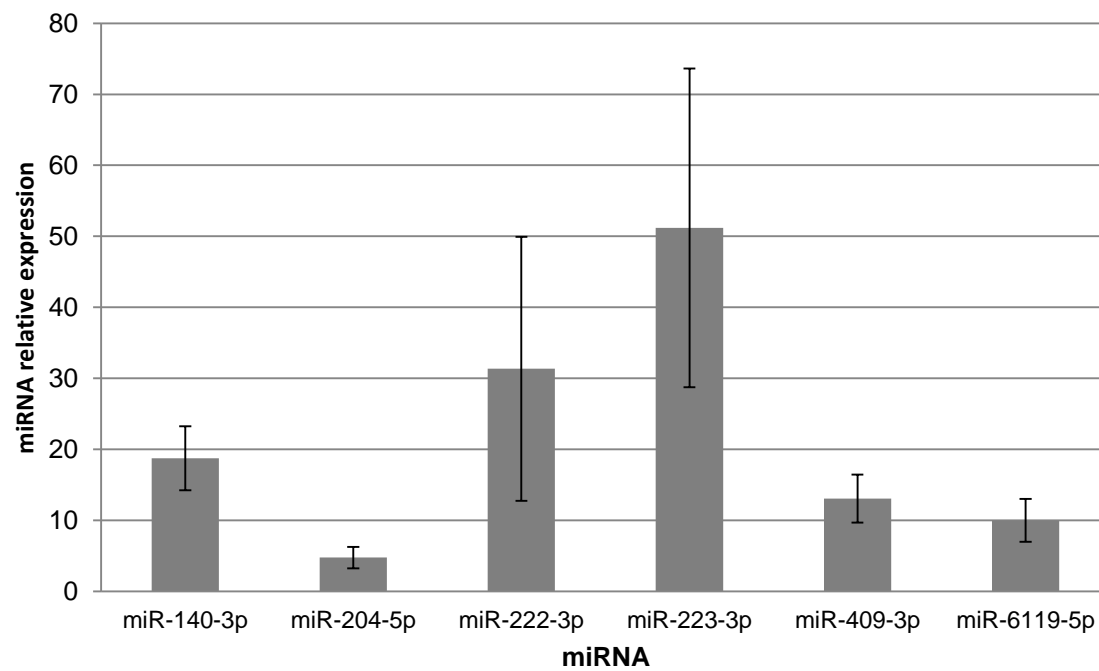

Supplement: Additional file 6: Figure S2. — Quantitative RT-PCR validation of NGS data. [file 12864_2015_1471_MOESM6_ESM.pdf]
