# Supplementary material for: Annotation of the goat genome using next generation sequencing of microRNA expressed by the lactating mammary gland: comparison of three approaches
Source: BMC Genomics. 2015 Apr 11;16(1):285. doi: 10.1186/s12864-015-1471-y (PMC4430871; doi:10.1186/s12864-015-1471-y)

Chromosomal localisation of miRNA (CHI1 to CHI10)

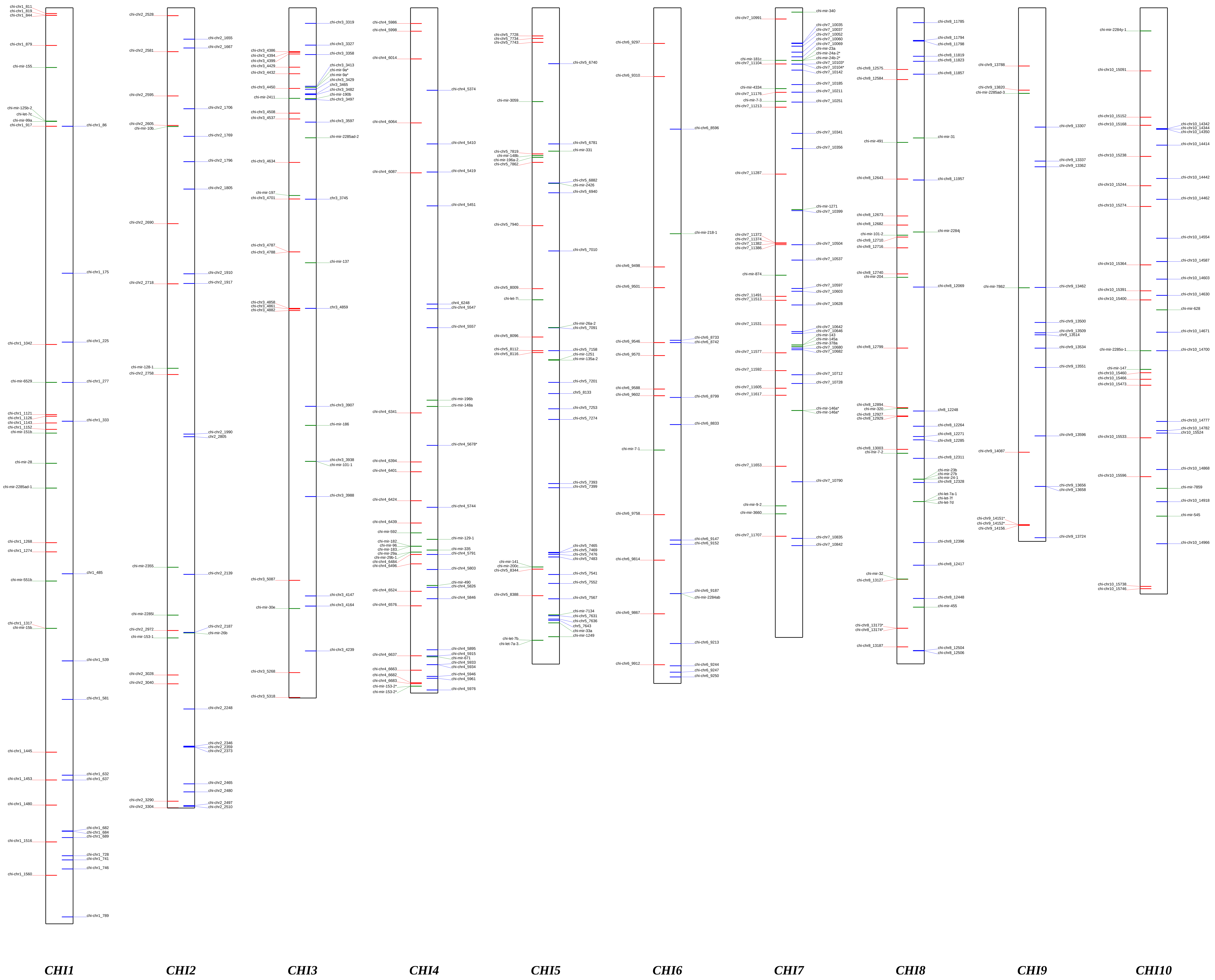

Chromosomal localisation of miRNA (CHI11 to CHI20)

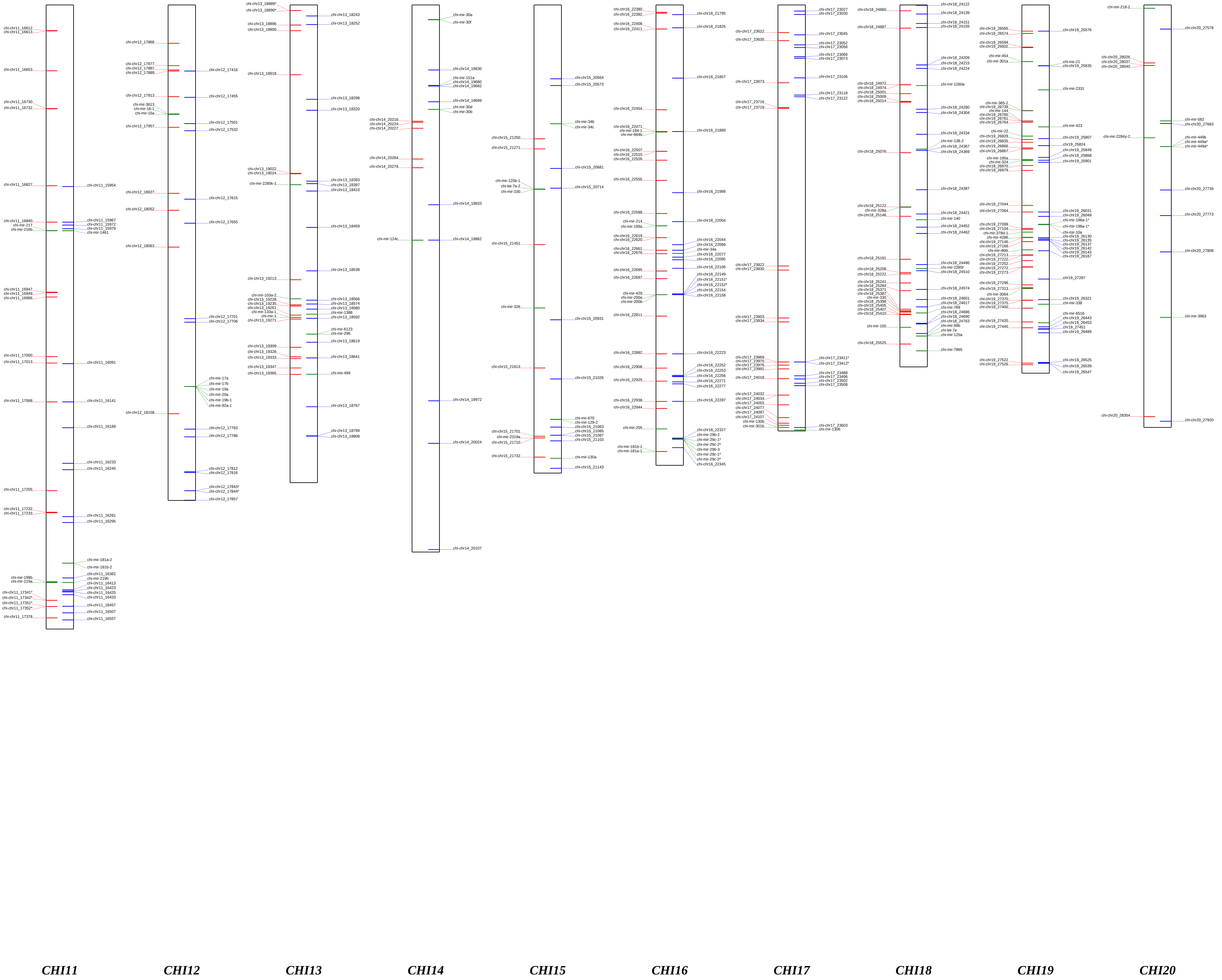

Chromosomal localisation of miRNA (CHI21 to CHI29 + CHIX)

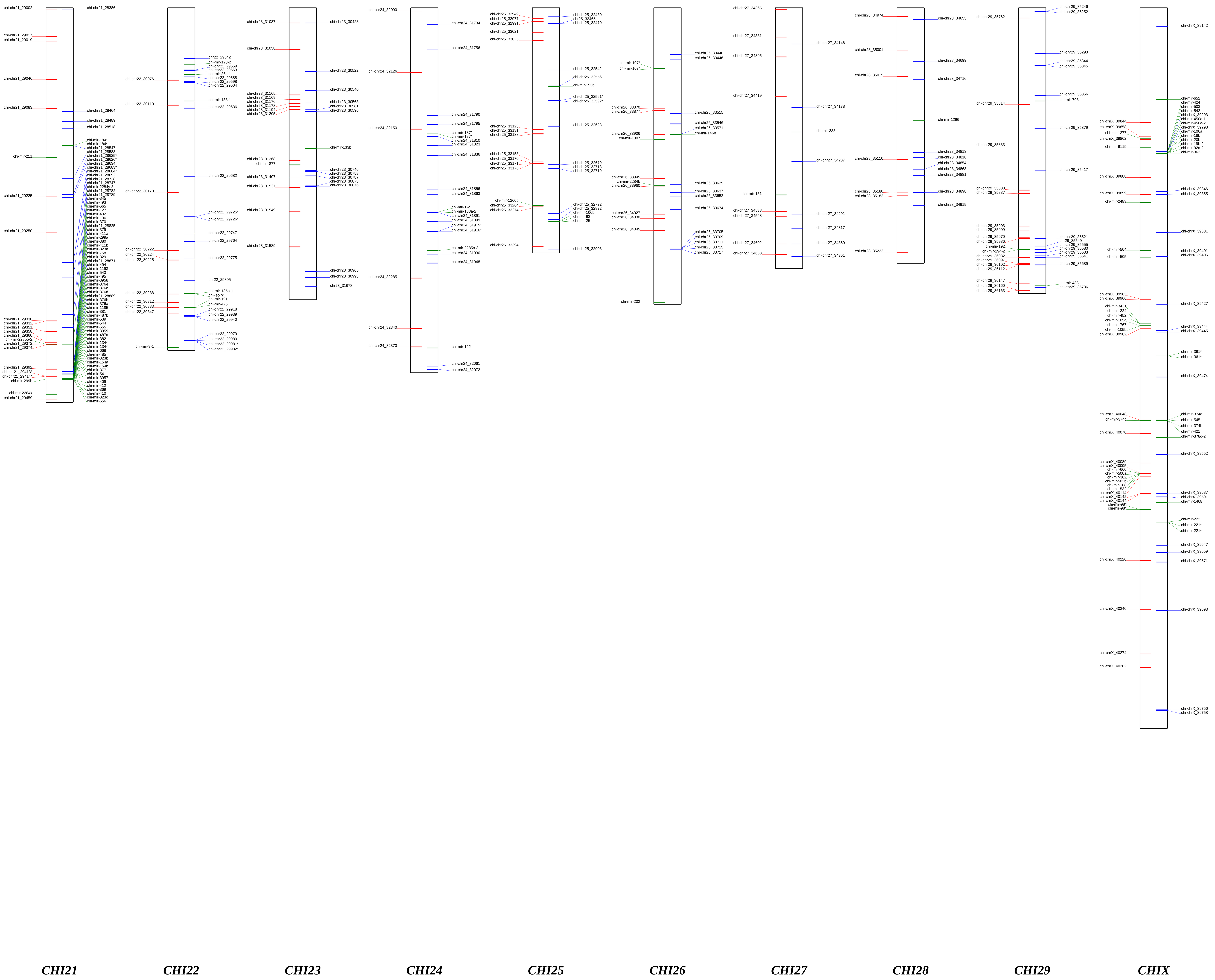

Supplement: Additional file 7: Figure S3. — Map of miRNA precursors on the goat genome CHIR_1. At the left of the chromosome, putative (red) and known (green) precursors localized on the + strand,. At the right of the chromosome, putative (blue) and known (green) precursors localized on the – strand. [file 12864_2015_1471_MOESM7_ESM.pdf]
